# Genomic characterization of *Klebsiella pneumoniae* clinical isolates from cancer patients: resistance profiles, virulence factors, and sequence typing

**DOI:** 10.3389/fmicb.2025.1676614

**Published:** 2025-10-31

**Authors:** Hancong Liu, Wan Liu, Xiaojuan Zhou, Xiaoli Wang, Gang Huang

**Affiliations:** Clinical Laboratory, Ganzhou Cancer Hospital, Ganzhou, Jiangxi, China

**Keywords:** malignant tumor, *Klebsiella pneumoniae*, antibiotic resistance, virulence gene, whole genome sequencing

## Abstract

**Background:**

*Klebsiella pneumoniae* (*K. pneumoniae*) is a major opportunistic pathogen associated with nosocomial infections, particularly in immunocompromised patients, including those with malignant cancer. The molecular epidemiology of *K. pneumoniae* isolated from distinct body sites from cancer patients remains poorly understood. This study aims to investigate the resistance genes, virulence factors, and sequence types of *K. pneumoniae* strains isolated from cancer patients to provide insights into their epidemiological and clinical characteristics.

**Methods:**

A total of 105 *K. pneumoniae* isolates from malignant cancer patients were subjected to whole-genome sequencing. Resistance genes, virulence- associated genes, and multilocus sequence typing (MLST) were analyzed. The distribution of these genetic determinants was compared among isolates from distinct body sites from cancer patients. Whole-genome sequencing was performed on 105 *K. pneumoniae* isolates obtained from the blood and body fluids of patients with various cancers, including lung (*n* = 24), nasopharyngeal (*n* = 12), liver (*n* = 11), cervical (*n* = 8), and other cancer types (*n* = 50). The isolates were characterized in terms of antimicrobial resistance genes, virulence-associated genes, and MLST. Additionally, virulence was evaluated using a scoring system incorporating the virulence genes ybt, clb, and iuc.

**Results:**

Among the isolates, 41 exhibited resistance to trimethoprim-sulfamethoxazole, predominantly harboring *sul1*, *sul2*, and *dfrA* genes. Resistance to tobramycin and levofloxacin was mainly associated with *aadA16*, *aph(3″)-Ib*, and *AAC(6′)-Ib-cr* genes. Eight isolates were identified as carbapenem-resistant *K. pneumoniae* (CRKP), carrying resistance genes *bla_kpc-1*, *bla_ndm-5*, and *bla_oxa-10*. Virulence genes included iron siderophores (*entAB*, *ybt*, *iucABC*), fimbriae (*fimA*, *fimH*), and *OmpA* (100%). Notably, isolates from cervical cancer patient samples had the lowest virulence scores, whereas those from lung and nasopharyngeal cancer patient samples demonstrated the highest virulence scores. MLST revealed 45 sequence types, with ST23 predominating in isolates from lung and nasopharyngeal cancer patient, while ST45 was the most frequent in cervical cancer patient isolates. Phylogenetic analysis demonstrated clustering of *Klebsiella pneumoniae* isolates from lung, nasopharyngeal, liver, and cervical cancer patient samples, with these isolates predominantly located within the third clade, accounting for 58.3, 66.7, 80, and 87.5%, respectively.

**Conclusion:**

The *K. pneumoniae* isolates in this study demonstrate considerable diversity in their virulence genes, antimicrobial resistance genes, and sequence types. The findings highlight the importance of genomic surveillance to guide infection control and therapeutic strategies, particularly in high-risk oncology settings.

## 1 Introduction

*Klebsiella pneumoniae* is a significant pathogen responsible for severe infections in cancer patients, particularly those with compromised immune systems due to chemotherapy or immunosuppressive therapy (Chen et al., 2024; Rolston, 2017). Infections caused by *K. pneumoniae* in this vulnerable population often lead to increased morbidity and mortality, with higher risks of severe complications compared to non-cancer patients (Nanayakkara et al., 2021). Studies have shown that cancer patients are more susceptible to *K. pneumoniae* infections, including bloodstream infections, respiratory tract infections, and urinary tract infections, which are often difficult to treat due to the emergence of multidrug-resistant strains (Navon-Venezia et al., 2017). The widespread use of antibiotics in cancer patients for infection prevention and treatment has accelerated the development of antibiotic resistance in *K. pneumoniae*. Multidrug-resistant *Klebsiella pneumoniae* (MDR-KP) and CRKP have emerged as major global public health threats. MDR-KP employs a range of mechanisms to confer resistance to commonly used antibiotics, including β-lactams, aminoglycosides, fluoroquinolones, and even agents of last resort such as polymyxins and tigecycline (Li et al., 2023). The resistance mechanisms are complex and involve the production of inactivating enzymes (e.g., Extended-Spectrum Beta-Lactamases (ESBLs), AmpC, and carbapenemases), loss or mutation of outer membrane porins (e.g., OmpK35/36) that reduce antibiotic uptake, overexpression of efflux pumps (e.g., AcrAB-TolC, OqxAB), modifications of antibiotic target sites, and the formation of protective barriers like biofilms (Karampatakis et al., 2023). Carbapenem resistance is chiefly mediated by carbapenemases, including *bla_kpc*, *bla_ndm*, *bla_vim*, *bla_imp*, and *bla_oxa-48* types, whose prevalence exhibits substantial geographic variation. For instance, in China, *bla_kpc-2* is the dominant carbapenemase gene in CRKP and is significantly more prevalent than other carbapenemase genes, such as bla_ndm and *bla_oxa-48* (Liao et al., 2022; Ge et al., 2024; Li et al., 2022). In contrast, bla_ndm-type metallo-β-lactamases are highly endemic in regions like South Asia, while *bla_oxa-48* and its variants predominate in Europe and North Africa, often linked to high-risk clones including ST101 and ST405 (Budia-Silva et al., 2024). Infections caused by CRKP are associated with high mortality, increased treatment failure, and elevated healthcare costs. Effective therapeutic options are scarce, especially for critically ill or immunocompromised patients. Treatment often depends on novel β-lactam/β-lactamase inhibitor combinations (e.g., ceftazidime-avibactam) or polymyxin-based regimens. Nonetheless, the ongoing evolution of resistance poses a persistent challenge (Karampatakis et al., 2023). Beyond carbapenemase production, additional mechanisms—such as efflux pump overexpression, porin loss, and biofilm formation—collectively promote carbapenem resistance, further constraining clinical treatment options (Ma et al., 2023). The high prevalence and increasing multidrug resistance of *K. pneumoniae* in clinical oncology practice are of considerable concern (Nanayakkara et al., 2021). A study by Amanati et al. (2021) reported that *K. pneumoniae* accounted for 14.5% of Gram-negative bacteria isolated from bloodstream infections in cancer patients; among these isolates, 60.7% exhibited an ESBLs-positive phenotype and 13.2% were CRKP, underscoring a substantial antimicrobial resistance burden in this patient population. Infections caused by hypervirulent *K. pneumoniae* (hvKp) are also alarming. One documented case described a postoperative rectal cancer patient who succumbed to liver infarction and septic shock following hvKp infection, highlighting the potential for rare yet fatal outcomes in immunocompromised hosts (Li et al., 2024). Additionally, strains harboring the pks genomic island—which encodes the genotoxin colibactin—have been reported in cancer patients. According to Wang et al. (2025), pks-positive strains accounted for 12.54% of isolates from Chinese cancer patients, with many belonging to hypervirulent clones such as ST23 and K1, suggesting a possible role for genotoxicity in both infection progression and oncogenesis. Oncology and intensive care units are recognized high-risk environments for the transmission of *K. pneumoniae*. Notably, CRKP has been detected in up to 59% of pediatric cancer patients in some studies, with evidence of clonal dissemination of strains carrying carbapenemase genes such as *bla_oxa-48* and *bla_ndm-1* (Osama et al., 2021). Among patients with hematological malignancies, ESBLs-producing strains are predominantly of the *bla_ctx-m* genotype (91%) and frequently display a multidrug-resistant phenotype (Lubwama et al., 2024).

In addition to antibiotic resistance, the virulence of *K. pneumoniae* plays a crucial role in its pathogenicity in cancer patients. *K. pneumoniae* possesses various virulence factors, including fimbriae (e. g., *fimA* and *fimH*) that enhance adhesion to host cells, and siderophores (e. g., *entA* and *entB*) that facilitate iron acquisition under host immune pressure (Choby et al., 2020). These virulence factors allow *K. pneumoniae* to effectively colonize, invade, and survive in the host, leading to severe and persistent infections (Mendes et al., 2023). Cancer patients, due to their immunocompromised state, are particularly vulnerable to the effects of these virulence mechanisms (Mishra et al., 2024).

While there has been extensive research on *K. pneumoniae* in general populations, studies focusing on its genomic and molecular characteristics in cancer patients remain limited. Given the unique immune status and treatment regimens of cancer patients, *K. pneumoniae* strains isolated from this group might harbor specific strain characteristics worthy of investigation (Ntim et al., 2025; Touati et al., 2020). Understanding these features is essential for guiding targeted treatment strategies and developing effective infection control measures. This study aims to investigate the genomic characteristics of *K. pneumoniae* isolated from cancer patients, focusing on antibiotic resistance genes and virulence determinants. By utilizing whole-genome sequencing, we seek to identify the genetic basis underlying the resistance and virulence of *K. pneumoniae* in this population, providing insights into its molecular epidemiology. The findings will contribute to a better understanding of the pathogen’s behavior in immunocompromised hosts and support the development of precision medicine approaches for managing *K. pneumoniae* infections in cancer patients.

## 2 Methods and materials

### 2.1 Bacterial isolation and identification

From October 2023 to September 2024, a total of 105 *K. pneumoniae* clinical isolates were obtained from the Department of Clinical Laboratory, Ganzhou Cancer Hospital. These isolates were derived from clinical specimens collected from 105 patients with various malignancies. The specimen types included sputum (48.6%), secretions (15.2%), urine (9.5%), blood cultures (6.7%), throat swabs (7.6%), and others (detailed in Supplementary material 1). The *K. pneumoniae* isolates incorporated in this study were selected based on the following criteria: (1) obtained from patients diagnosed with primary malignant tumors; (2) represented the first obtained isolate from each individual patient; (3) Serum inflammatory markers exceeding the following thresholds: C-reactive protein (CRP) > 10 μg/mL or procalcitonin (PCT) > 0.5 ng/mL. The isolates were identified as *K. pneumoniae* using matrix-assisted laser desorption ionization-time of flight mass spectrometry MALDI-TOF/MS (America, BEXS2600). Susceptibility testing was conducted using the AST-N334 card on the VITEK 2 Compact automated system (bioMérieux, France). This card tests the following antibiotics across the indicated concentration ranges: Amikacin (2–64 μg/mL), Amoxicillin/Clavulanic Acid (2/1–32/16 μg/mL), Cefepime (0.12–32 μg/mL), Cefoperazone/Sulbactam (8–64 μg/mL), Cefoxitin (4–64 μg/mL), Ceftazidime (0.12–64 μg/mL), Ceftriaxone (0.25–64 μg/mL), Cefuroxime (1–64 μg/mL), Ertapenem (0.12–8 μg/mL), Imipenem (0.25–16 μg/mL), Levofloxacin (0.12–8 μg/mL), Piperacillin/Tazobactam (4/4–128/4 μg/mL), Tigecycline (0.5–8 μg/mL), and Trimethoprim/Sulfamethoxazole (1/19–16/304 μg/mL). Furthermore, the Kirby-Bauer disk diffusion method was also performed with disks containing: Cefotaxime (30 μg), Ampicillin (10 μg), Ampicillin/Sulbactam (10/10 μg), Tobramycin (10 μg), Aztreonam (30 μg), and Cefazolin (30 μg). All isolates were cultured on blood agar plates, and antibiotic susceptibility tests were performed according to the Clinical and Laboratory Standards Institute 2024 guidelines. All procedures were carried out and results were interpreted based on the standards provided by Clinical and Laboratory Standards Institute (2024). *Escherichia coli* ATCC25922 was used as the quality control strain.

### 2.2 String test

A string test was performed to identify hypermucoviscous phenotypes. Bacterial colonies were grown overnight on blood agar plates at 37 °C. A loop was used to stretch a colony, and strains were considered positive for hypermucoviscosity if a viscous string >5 mm in length was observed (Kumabe and Kenzaka, 2014).

### 2.3 Whole-genome sequencing

The whole-genome sequencing of the *K. pneumoniae* isolates was performed to identify the distribution of antibiotic resistance genes and virulence factors. Colonies were inoculated into LB liquid medium (components: Tryptone (Oxoid, UK; manufactured in France), Yeast Extract (Oxoid, UK; manufactured in the United Kingdom), and Sodium Chloride [Macklin, China]). and incubated at 37 °C for 16 h, followed by centrifugation at 8,000 rpm for 10 min to collect bacterial pellets. Total DNA was extracted using a commercial kit (Beyotime, D0063) according to the manufacturer’s protocol. DNA purity was assessed with a NanoPhotometer® spectrophotometer (IMPLEN, USA), while concentration was quantified using a Qubit® 3.0 Fluorometer (Life Technologies, USA). DNA integrity and contamination were evaluated via 1% agarose gel electrophoresis (120 V, 45 min). Qualified samples (0.5 μg gDNA per reaction) underwent library preparation with the Annoroad® Universal DNA Fragmentase Kit v2.0 and Library Prep Kit v2.0 (AN200101-L) following standardized protocols. Cluster generation was performed on cBot using HiSeq PE Cluster Kit v4-cBot-HS (Illumina), and paired-end sequencing (150 bp) was conducted on the HiSeq platform with HiSeq X Ten Reagent Kit v2.5. Base calling was executed via WriteFQ software to generate raw sequencing reads (Sequenced Reads). Virulence factor genes were predicted by BLASTP analysis of the annotated protein sequences against the core dataset of the Virulence Factors Database (VFDB). Antimicrobial resistance genes were annotated using the Resistance Gene Identifier (RGI) tool against the Comprehensive Antibiotic Resistance Database (CARD), with a minimum identity cutoff of 80%. Sequence types were determined using the web-based PubMLST typing tool.^1^ Phylogenetic trees were constructed using the maximum likelihood method in MEGA11 software, based on multiple sequence alignments.

### 2.4 Virulence scoring

Virulence scoring was performed according to the system established by Lam et al. (2021), which assigns a quantitative virulence score based on the detection of three key virulence determinants: yersiniabactin (ybt), colibactin (clb), and aerobactin (iuc). The scoring system is designed to reflect increasing clinical risk associated with specific genetic profiles: a score of 0 indicates absence of all three genes; 1 indicates presence of ybt only (a siderophore linked to immune evasion but considered moderately virulent alone); 2 indicates presence of clb without iuc (irrespective of ybt, reflecting the genotoxic potential of clb); 3 indicates presence of iuc only (a hallmark of virulence plasmids associated with severe infections such as sepsis); 4 indicates coexistence of iuc and ybt in the absence of clb; and 5 indicates the presence of all three genes, representing the highest-risk profile. This hierarchical framework integrates established clinical and molecular insights into *Klebsiella pneumoniae* pathogenicity, translating complex genomic data into an interpretable and reproducible numerical score that facilitates consistent cross-study comparisons and enhances evaluative clarity.

### 2.5 Statistical analysis

Data visualization and statistical analyses were performed using GraphPad Prism 8.0. Quantitative data were expressed as mean ± standard deviation. Categorical data were presented as percentages (%), and comparisons were made using the Chi-square test or Fisher’s exact test. A *p*-value of <0.05 was considered statistically significant.

## 3 Results

### 3.1 Clinical characteristics of *K. pneumoniae* isolates

A total of 105 non-duplicate *K. pneumoniae* clinical isolates were obtained from patient specimens. Detailed clinical information is presented in Table 1. The majority of isolates were collected from patients with lung malignancies (22.9%, *n* = 24), followed by nasopharyngeal malignancies (11.4%, *n* = 12), liver malignancies (10.5%, *n* = 11), and cervical malignancies (7.6%, *n* = 8). The majority of isolates (85.7%, 90/105) were collected from specialized medical and surgical oncology wards, while a smaller proportion originated from the intensive care unit (7.6%, 8/105) and radiotherapy wards (9.5%, 10/105). The distribution of specimen types varied by malignancy type (Table 2). For lung malignancy patients, sputum was the predominant specimen type (83.3%). Similarly, sputum accounted for the majority of samples from nasopharyngeal malignancy patients (75.0%). In contrast, isolates from liver malignancy patients were primarily obtained from drainage fluid (27.3%), while cervical malignancy patients had a high proportion of isolates from secretions (62.5%).

**Table 1:** Clinical and demographic information of *K. pneumoniae* infection cases.

Items	No. of strains (*n*)	Proportion
Gender		
Male	74	70.50%
Female	31	29.50%
Age	61	–
Specimen type		
Sputum	51	48.60%
Urine	10	9.50%
Blood culture	7	6.70%
Secretions^a^	16	15.20%
Peritoneal fluid	4	3.80%
Pharyngeal swab	8	7.60%
PICC tube	1	1.00%
Pus	1	1.00%
Drainage fluid^b^	3	2.90%
Irrigation fluid^c^	4	3.80%
Disease type		
Cervical malignancy	8	7.60%
Esophageal malignancy	6	5.70%
Rectal malignancy	5	4.80%
Lung malignancy	24	22.90%
Liver malignancy	11	10.50%
Brain malignancy	4	3.80%
Gastric malignancy	4	3.80%
Nasopharyngeal malignancy	12	11.40%
Skin malignancy	3	2.90%
Pancreatic malignancy	2	1.90%
Colon malignancy	2	1.90%
Lymphoma	3	2.90%
Other malignancies	18	17.10%
Ovarian malignancy	3	2.90%
Departments		
Department of Critical Care Medicine	8	7.6%
Medical Ward for Chest Oncology	12	11.4%
Radiotherapy Ward for Chest Oncology	2	1.9%
Department of Thoracic Surgery	10	9.5%
Department of Head and Neck Surgery	6	5.7%
Radiotherapy Ward for Head Oncology	16	15.2%
Ward for Lymphoma and Hematologic Oncology	11	10.5%
Department of Interventional Therapy	2	1.9%
Department of Emergency and General Medicine	7	6.7%
Radiotherapy Ward for Abdominopelvic Oncology	8	7.6%
Medical Ward for Abdominal Oncology	10	9.5%
Department of Abdominal Surgery	4	3.8%
Department of Gynecologic Oncology	5	4.8%
Palliative and Hospice Care Ward	4	3.8%

a Secretions are defined as pathological discharges that are spontaneously released from body tissues or organs.

b Drainage fluid refers to liquid collected from body cavities or tissue spaces via surgical drainage devices or natural drainage tracts.

c Irrigation fluid denotes liquid that is introduced into a body cavity or wound during a clinical procedure and subsequently retrieved.

**Table 2:** Specimen types of patients with different malignancy.

Specimen type	Lung malignancy, *n* = 24	Nasopharyngeal malignancy, *n* = 12	Liver malignancy, *n* = 11	Cervical malignancy, *n* = 8
Sputum	20	9	2	0
Secretions	0	1	1	5
Drainage fluid	0	0	3	0

### 3.2 Antibiotic susceptibility of *K. pneumoniae* isolates

**Figure 1:**
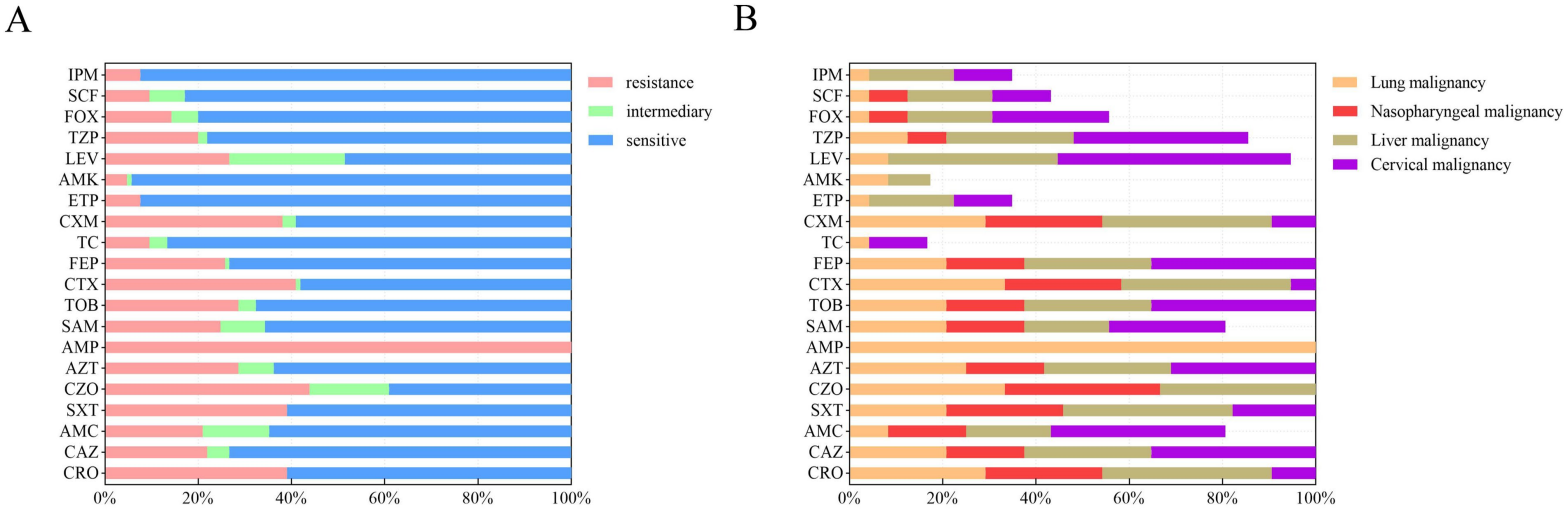
Antibiotic susceptibility of 105 *K. pneumoniae* isolates from patients with malignant tumors. **(A)** Overall resistance rates of all isolates to 20 antibiotics, presented as percentages. **(B)** Resistance rates of *K. pneumoniae* isolates stratified by four major cancer types: lung (*N* = 24), nasopharyngeal (*N* = 12), liver (*N* = 11), and cervical (*N* = 8) malignancies. Rates reflect the proportion of resistant isolates within each cancer group. IPM, imipenem; SCF, cefoperazone/sulbactam; FOX, cefoxitin; TZP, piperacillin/tazobactam; LEV, levofloxacin; AMK, amikacin; ETP, ertapenem; CXM, cefuroxime; TC, tigecycline; FEP, cefepime; CTX, cefotaxime; TOB, tobramycin; SAM, ampicillin/sulbactam; AMP, ampicillin; AZT, aztreonam; CZO, cefazolin; SXT, trimethoprim-sulfamethoxazole; AMC, amoxicillin/clavulanic acid; CAZ, ceftazidime; CRO, ceftriaxone.

The antibiotic susceptibility of 105 *K. pneumoniae* isolates to 20 antibiotics was determined using broth microdilution (Figure 1A). Resistance was observed for all tested antibiotics. The overall resistance rates for key antibiotics were as follows: ceftriaxone (39.0%, *n* = 41), ceftazidime (22.9% *n* = 23), trimethoprim-sulfamethoxazole (39.0%, *n* = 41), cefazolin (43.8%, *n* = 46), aztreonam (28.6%, *n* = 30), ampicillin (100%, *n* = 105), ampicillin-sulbactam (24.8%, *n* = 26), tobramycin (28.6%, *n* = 30), cefotaxime (41.0%, *n* = 43), cefepime (25.7%, *n* = 27), cefuroxime (38.1%, *n* = 40), levofloxacin (26.7%, *n* = 28), and piperacillin-tazobactam (20.0%, *n* = 21).

Resistance patterns varied among isolates from patients with different cancer types (Figure 1B). Lung cancer patient samples: High resistance rates were observed for cefazolin (33%, *n* = 8), cefotaxime (33%, *n* = 8), and aztreonam (25%, *n* = 6). Nasopharyngeal cancer patient samples: Isolates exhibited notable resistance to cefazolin (33.3%, *n* = 4), trimethoprim- sulfamethoxazole (25%, *n* = 3), and aztreonam (16.7%, *n* = 2). Liver cancer patient samples: Resistance was highest for trimethoprim-sulfamethoxazole (36.4%, *n* = 4), cefotaxime (36.4%, *n* = 4), and levofloxacin (36.4%, *n* = 4). Cervical cancer patient samples: Isolates showed high resistance to cefotaxime (75%, *n* = 8), levofloxacin (50%, *n* = 4), and aztreonam (50%, *n* = 4). Across all malignancy types, resistance rates to cephalosporins were consistently >30%. Notably, isolates from liver and cervical malignancy patients exhibited ≥40% resistance to levofloxacin.

### 3.3 Whole-genome sequencing results

The quality of the raw sequencing data was assessed using FastQC. Adapters and low-quality bases were trimmed with Trimmomatic to ensure high-quality data for downstream analysis.

#### 3.3.1 Antibiotic resistance genes

Whole-genome sequencing was performed on 105 *K. pneumoniae* isolates, and the distribution of antibiotic resistance genes is summarized in Table 3. Among the 41 trimethoprim-sulfamethoxazole-resistant isolates, the following resistance genes were identified: *sul1* (70%), *sul2* (80%), *sul3* (7.5%), *dfrA1* (2.5%), *dfrA12* (27.5%), *dfrA14* (32.5%), *dfrA17* (2.5%), and *dfrA27* (52.5%). Co-occurrence of resistance genes was noted in 19 isolates harboring *sul1* + *sul2* (48%) and 2 isolates carrying *sul1* + *sul2* + *sul3* (5%).

**Table 3:** Distribution of drug and virulence genes in 105 *K. pneumoniae* strains.

Resistance genes	Strains	Proportion	Virulence genes	Strains	Proportion
Aminoglycoside (*n* = 30)			Siderophore aerobactin (*n* = 105)		
*aadA16*	17	56.70%	*iucA*	52	50%
*aadA2*	11	36.70%	*iucB*	53	51.10%
*aph (3′)-Ia*	16	53.30%	*iucC*	51	49.00%
*aph (3″)-Ib*	18	60.00%	*iutA*	51	49.00%
*aph (4)-Ia*	8	26.70%	Siderophore enterobactin (*n* = 105)		
*aph (6)-Id*	18	60.00%	*entA*	94	90.40%
*ACC (3)-IId*	13	43.30%	*entB*	104	100%
*ACC (3)-IIV*	8	26.70%	*fepC*	102	98.10%
Sulfonamide (*n* = 41)			*fepG*	9	8.70%
*sul1*	28	68.3%	Siderophore Salmonella chela (*n* = 105)		
*sul2*	32	78.0%	*iroB*	42	40.40%
*sul3*	3	7.3%	*iroC*	41	39.40%
*sul1 + sul2*	19	46.3%	*iroD*	42	40.40%
*sul1 + sul2 + sul3*	2	4.9%	*iroN*	42	40.40%
*dfrA1*	1	2.4%	Siderophore Yersinia chela (*n* = 105)		
*dfrA12*	11	26.8%	*irp1*	60	57.7%
*dfrA14*	13	31.7%	*irp2*	63	60.6%
*dfrA17*	1	2.4%	*fyuA*	62	59.6%
*dfrA27*	21	51.2%	*ybtA*	63	60.6%
Quinolone (*n* = 28)			*ybtE*	62	59.6%
*AAC (6′)-Ib-cr*	21	75.00%	*ybtP*	62	59.6%
*QnrB17*	6	21.40%	*ybtQ*	62	59.6%
*QnrB20*	6	21.40%	*ybtS*	63	60.6%
*QnrB4*	5	17.90%	*ybtT*	65	62.50%
*QnrS1*	17	60.70%	*ybtU*	62	59.60%
Extended-spectrum β-lactamase (*n* = 39)			*ybtX*	62	59.6%
*CTX-M*	34	87.20%	Fimbriae (*n* = 105)		
*SHV*	7	17.90%	*MrkD*	1	1.00%
*TEM*	28	71.80%	*FimA+mrkD*	2	1.90%
*DHA-1*	7	17.90%	*FimA+mrkD+fimH*	96	92.30%
*LAP-2*	4	10.30%	Other (*n* = 105)		
*CTX+SHV*	8	20.50%	*astA*	12	11.50%
*CTX+TEM*	1	2.50%	*cseA*	14	13.50%
*SHV+TEM*	6	15.40%	*mgtB*	1	1.00%
*CTX-M + SHV+TEM*	18	46.20%	*ompA*	100	96.20%
Carbapenem (*n* = 8)			String test (*n* = 105)		
*bla_kpc-1*	2	25.0%	Positive	37	35.20%
*bla_oxa-10*	1	12.5%	Negative	68	64.80%
*bla_ndm-5*	2	25.0%			
Drug-resistant phenotype (*n* = 105)					
*ESBLs Positive*	39	37.10%			
*ESBLs Negative*	66	62.90%			
*MDR-KP*	45	42.90%			
*Non-MDR-KP*	60	57.10%			

**Figure 2:**
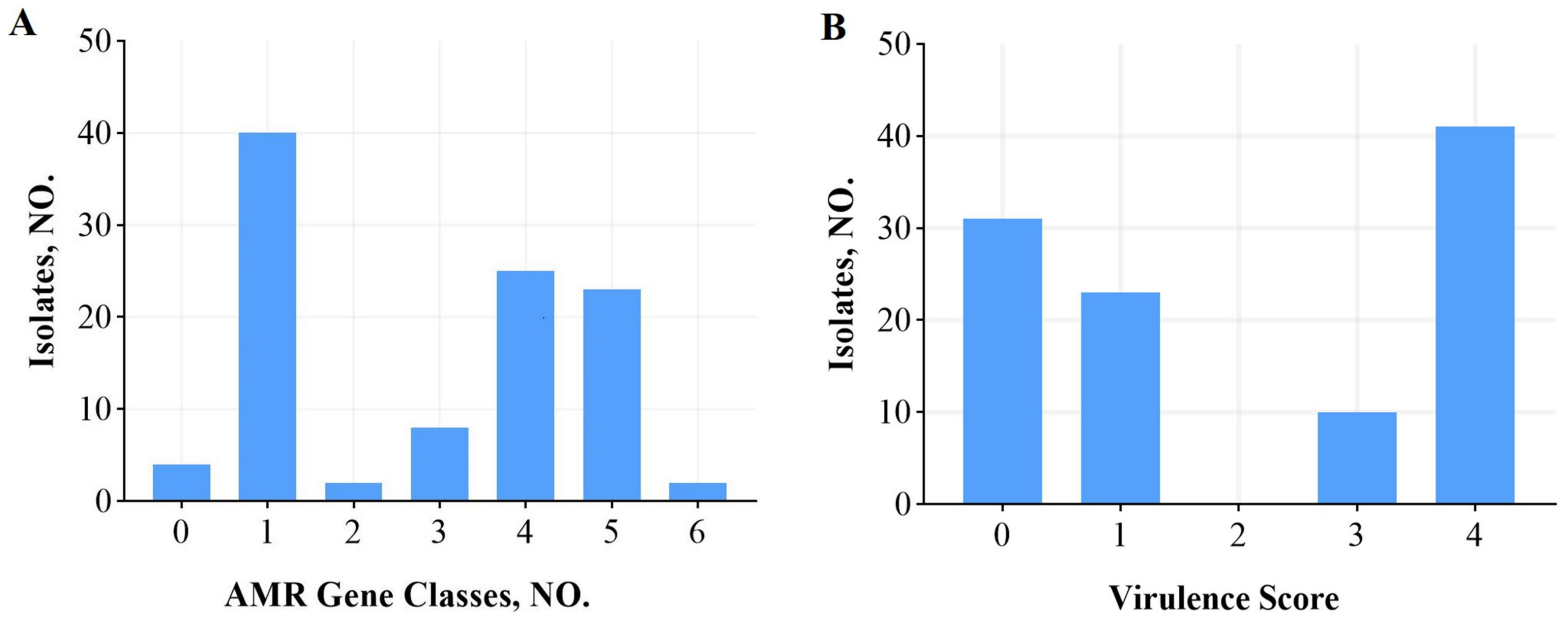
AMR gene categories and virulence scores among 105 *K. pneumoniae* isolates. **(A)** Number of antibiotic resistance gene categories per isolate. **(B)** Virulence scores (0–5) assigned using the Lam system based on the presence of yersiniabactin (ybt), colibactin (clb), and aerobactin (iuc) genes. A score ≥ 3 indicates high virulence potential. Nearly half of all isolates (48%) scored ≥3. The proportion of highly virulent isolates (score ≥ 3) varied across malignancy types. AMR, antimicrobial resistance.

Among the 30 isolates resistant to tobramycin, the predominant genes included *aadA16* (56.7%), *aadA2* (36.7%), *aph(3′)-Ia* (53.3%), *aph(3″)-Ib* (60%), *aph(6)-Id* (60%), *aph(4)-Ia* (26.7%), *ACC(3)-IId* (43.3%), and *ACC(3)-IIV* (26.7%). In the 28 isolates resistant to levofloxacin, the primary resistance genes identified were *AAC(6′)-Ib-cr* (75%), *QnrS1* (60.7%), *QnrB17* (21.4%), *QnrB20* (21.4%), and *QnrB4* (17.9%). CRKP was observed in 8 isolates, with harboring *bla_kpc-1* (40%), *bla_ndm-5* (40%), and *bla_oxa-10* (20%). Among 39 extended-spectrum beta-lactamase (ESBLs)-producing isolates, the most common genes were *CTX-M* (87.2%), *SHV* (94.9%), *TEM* (71.8%), *DHA-1* (17.9%), and *LAP-2* (10.3%), with co-occurrence patterns including *CTX-M* + *SHV* (8 isolates), *CTX-M* + *TEM* (1 isolate), *SHV* + *TEM* (6 isolates), and *CTX-M* + *SHV* + *TEM* (18 isolates). Efflux pump genes were frequently detected, including *acrB* (100%), *acrD* (97.1%), *acrA* (100%), *oqxB* (97.1%), *oqxA* (99.0%), *marA* (100%), and *ramA* (100%). A detailed analysis showed that 40 isolates carried a single resistance gene, 25 carried four, and 23 carried five, with more than three resistance genes detected in 53.8% of isolates (Figure 2A).

The distribution of resistance genes varied across isolates from patients with different cancer types, as shown in Table 4. For quinolone resistance, isolates from lung, nasopharyngeal, liver, and cervical cancer patient samples predominantly harbored *AAC(6′)-Ib-cr* and *QnrS1*, with the highest prevalence of *AAC(6′)-Ib-cr* observed in cervical cancer patient samples. For aminoglycoside resistance, *APH(3″)-Ib* and *APH(6)-Id* were dominant, with cervical cancer patient samples isolates showing the highest frequency of aminoglycoside resistance genes (≥25%). For sulfonamide resistance, *sul1* and *sul2* were predominant, with nasopharyngeal cancer patient samples isolates most frequently carrying *dfrA14* and *sul2* (25%). For ESBLs genes, *TEM-1* was most prevalent, with cervical cancer patient samples isolates showing the highest proportion of *TEM-1* (62.5%).

**Table 4:** Resistance genes, virulence genes and virulence scores of different *K. pneumoniae* isolates.

Items	Lung malignancy (*N* = 24)	Nasopharyngeal malignancy (*N* = 12)	Liver malignancy (*N* = 11)	Cervical malignancy (*N* = 8)	*p*-value
Antibiotic resistance genes					
*AAC(6′)-Ib-cr*	12.50%	8.30%	18.18%	62.50%	0.020^d^
*QnrS1*	37.50%	33.30%	27.27%	25.00%	0.931
*APH(3″)-Ib*	37.50%	25.00%	36.36%	12.50%	0.573
*APH(6)-Id*	41.70%	25.00%	36.36%	25.00%	0.763
*sul1*	33.30%	16.70%	36.36%	62.50%	0.237
*sul2*	37.50%	25.00%	36.36%	50.00%	0.728
*dfrA14*	8.30%	25.00%	9.09%	25.00%	0.407
*TEM-1*	25.00%	16.70%	18.18%	62.50%	0.134
Virulence genes					
*ybt*	75.00%	50.00%	36.40%	50.00%	0.126
*fyuA*	75.00%	50.00%	36.40%	50.00%	0.126
*iroBCDN*	58.30%	41.70%	36.40%	12.50%	0.138
*entB*	100.00%	100.00%	100.00%	87.50%	0.145
*entA*	95.80%	66.70%	72.70%	100.00%	0.030^d^
*iucABC*	54.20%	58.30%	54.50%	12.50%	0.173
*iutA*	54.20%	50.00%	54.50%	12.50%	0.210
*fimH*	100.00%	100.00%	100.00%	100.00%	N/A^f^
*fimA*	95.80%	100.00%	100.00%	100.00%	0.642
*mrkD*	100.00%	100.00%	100.00%	100.00%	N/A^f^
Virulence score					
0	17%	33%	55%	38%	0.134
1	29%	17%	0%	50%	0.052
2	0%	0%	0%	0%	N/A^f^
3	8%	8%	18%	0%	0.726
4	46%	42%	27%	13%	0.349

d Statistical findings indicate that the distribution of gene proportions is not uniform across the four malignancy types: lung, nasopharyngeal, hepatic, and cervical cancers.

f Unable to perform or not necessary to perform this statistical test.

#### 3.3.2 Virulence genes

Virulence gene profiles of the 105 *K. pneumoniae* isolates are summarized in Table 3, including siderophore and fimbrial genes such as *fimA*, *fimH*, and *markD*. The siderophore genes showed the following distribution: aerobactin (49%), yersiniabactin (32%), enterobactin (100%), and salmochelin (40%). A total of 96 isolates carried *fimA* + *fimH* + *markD*, 2 isolates carried *fimA* + *markD*, and 1 isolate carried only *markD*. The *ompA* gene was universally present (100%), while *astA*, *cseA*, and *mgtB* were less frequently detected.

Siderophore gene distribution varied among isolates from patients with different cancer types, as shown in Table 4. Isolates from nasopharyngeal cancer patient samples most frequently harbored the aerobactin-encoding *iucABC* genes, while lung cancer patient samples isolates showed the highest prevalence of yersiniabactin-related genes (*ybt* and *fyuA*). Conversely, cervical cancer patient samples isolates exhibited the lowest prevalence of *iucABC* and *iroBCDN* (salmochelin). Enterobactin genes (*entAB*) were present in ≥87.5% of isolates across all malignancies, with the highest prevalence of *iroBCDN* seen in lung cancer isolates.

Virulence scores, calculated using a system developed by Lam et al. that incorporates the number and type of virulence genes associated with clinical risk, ranged from 0 to 5. Scores ≥3, indicating high virulence potential, were found in 48% of isolates (Figure 2B). High scores (≥3) were more common in isolates from lung (50%), nasopharyngeal (50%), and liver cancer patients, whereas cervical cancer isolates showed the lowest prevalence of high virulence scores (13%).

#### 3.3.3 Sequence types

Whole-genome sequencing identified 45 sequence types among the isolates. The most common types were ST23 (15.3%), followed by ST45 (6.7%), ST37 (4.8%), and ST25 (3.8%). Isolates from lung and nasopharyngeal cancer patient samples were predominantly ST23, whereas ST45 was most common among cervical cancer patient samples isolates, accounting for 37.5%.

#### 3.3.4 Phylogenetic relationships and virulence gene analysis of *K. pneumoniae* isolates

**Figure 3:**
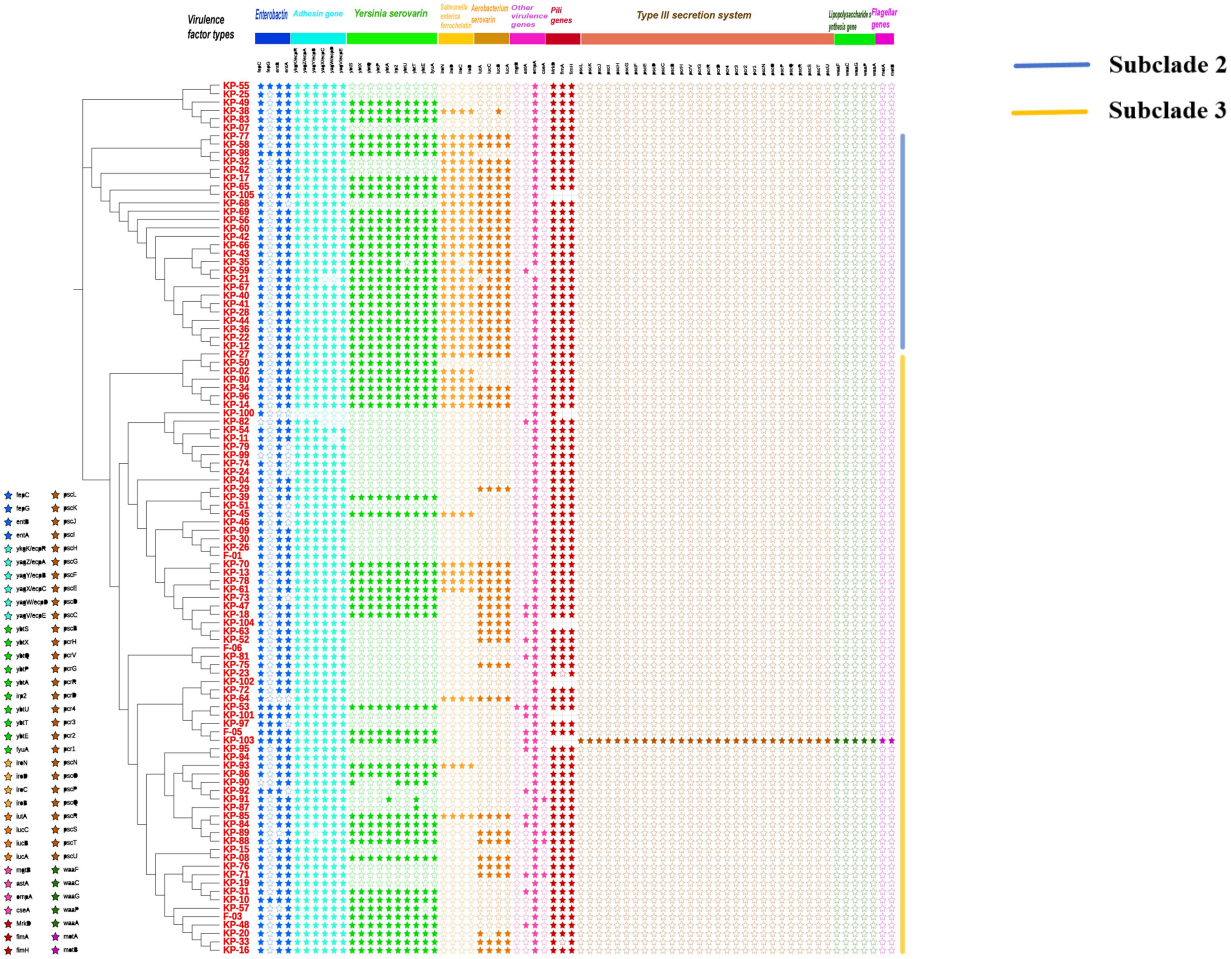
Phylogenetic relationships and virulence gene profiles of 105 *K. pneumoniae* isolates from patients with different malignant tumors. The maximum-likelihood phylogenetic tree (left) was constructed from whole-genome sequences. The accompanying heatmap (right) illustrates the distribution of key virulence genes across the strains. Genes are categorized by function. Symbols denote the presence (solid star, ★) or absence (hollow star, ☆) of each gene.

Whole-genome sequencing was performed on all *K. pneumoniae* isolates, and a core single nucleotide polymorphism-based phylogenetic tree was constructed (Figure 3). Virulence gene profiles of each isolate were analyzed, including genes encoding enterobactin, adhesins, yersiniabactin, salmochelin, aerobactin, other virulence factors, fimbrial proteins, key components of the type III secretion system, lipopolysaccharide synthesis, and flagellar proteins. The phylogenetic analysis revealed three main branches. Strains in the second branch exhibited 84.6% positivity in the string test but had a low prevalence of ESBLs production (3.8%). These strains carried the most diverse siderophore virulence genes. In contrast, strains in the third branch demonstrated 18.9% positivity in the string test but a significantly higher ESBLs positivity rate (45.9%). The third branch predominantly included isolates from patients with lung (58.3%), nasopharyngeal (66.7%), liver (80%), and cervical (87.5%) malignant tumors. The most prevalent virulence genes were those encoding enterobactin (*fepC*: 101/105, *entB*: 100/105, *entA*: 91/105), adhesins (*ykgK/ecpR*: 103/105, *yagZ/ecpA*: 103/105, *yagY/ecpB*: 102/105, *yagX/ecpC*: 101/105, *yagW/ecpD*: 100/105, *yagV/ecpE*: 102/105), and fimbrial proteins (*MrkD*: 98/98, *fimA*: 96/98, *fimH*: 98/98). One MDR isolate, K-103, was identified from a patient with hypopharyngeal carcinoma. This ESBLs-producing strain was unique in carrying a complete set of key virulence genes, including those related to the type III secretion system, lipopolysaccharide synthesis, and flagellar proteins.

## 4 Discussion

*Klebsiella pneumoniae* is a significant pathogen capable of colonizing the respiratory, gastrointestinal, and urinary tracts and causing invasive infections, particularly in immunocompromised patients with malignant tumors (Lam et al., 2021). Neutropenia in these patients often necessitates prolonged antibiotic therapy, leading to the emergence of highly resistant strains. These circumstances complicate chemotherapy and treatment outcomes. This study characterized 105 *K. pneumoniae* isolates from malignant tumor patients, focusing on resistance profiles, virulence factors, and sequence typing. The specimens were primarily collected from sputum, secretions, and urine. In contrast, Victoria Ballén et al. (Holt et al., 2015) identified urine as the most common source, with 84.21% of urinary isolates carrying the uge virulence gene. Our study’s predominance of sputum specimens likely reflects the high prevalence of pulmonary and nasopharyngeal malignancies and the frequent use of ventilators in advanced-stage patients, which increases the risk of pulmonary infections.

Antibiotic susceptibility testing revealed resistance to all 20 tested antibiotics, with isolates from lung, nasopharyngeal, liver, and cervical cancer patient samples exhibiting particularly high resistance rates (≥25%) to β-lactams, sulfonamides, and quinolones. These results align with earlier findings, which report widespread MDR in *K. pneumoniae*, including resistance to β-lactams, quinolones, aminoglycosides, and sulfonamides (Ballén et al., 2021). Similarly, Anderson Lineu Siqueira dos Santos et al. (Abdelwahab et al., 2021) observed high resistance rates to ampicillin (95.3%), cefuroxime (67.2%), gentamicin (32.8%), and trimethoprim-sulfamethoxazole (12.5%) in oncology patients, consistent with our observations. These findings emphasize the growing challenge of MDR *K. pneumoniae* in malignant tumor settings.

Whole-genome sequencing revealed multiple resistance and virulence genes among the isolates. Common resistance genes included *AAC(6′)-Ib-cr*, *QnrS1*, *APH(3″)-Ib*, *APH(6)-Id*, *sul1*, and *sul2*, conferring resistance to quinolones, aminoglycosides, and sulfonamides. Notably, cervical cancer patient samples isolates exhibited the highest prevalence of *AAC(6′)-Ib-cr* and aminoglycoside resistance genes. Santos et al. (2020) and Enany et al. (2022) also identified similar resistance mechanisms in *K. pneumoniae* ST627 strains, corroborating our findings. The study also identified eight CRKP isolates, with *bla_kpc-1* (40%), *bla_ndm-5* (40%), and *bla_oxa-10* (20%) being the predominant carbapenemase genes. These results differ from reports citing *bla_vim-1*, *bla_ndm-1*, *bla_oxa-48*, and *bla_kpc-2* as common carbapenemase genes (Karampatakis et al., 2023), possibly due to the limited number of CRKP isolates. Additionally, 39 isolates were ESBLs-positive, with most carrying *CTX-M* (87.2%) and *SHV* (94.9%) genes, consistent with findings by Binzhi Dan et al. (Ma et al., 2023), who reported high co-occurrence of *CTX-M*, *SHV*, and *TEM* genes.

Efflux pump genes, which contribute to antibiotic resistance, were highly prevalent among the isolates. Key efflux pump genes detected included *acrB* (100%), *acrD* (97.1%), *acrA* (100%), *oqxB* (97.1%), *oqxA* (99.0%), *marA* (100%), and *ramA* (100%). These findings align with studies showing that efflux pumps are integral to the MDR phenotype of *K. pneumoniae* (Dan et al., 2023). Amir Mirzaie and Reza Ranjbar (Ren et al., 2024) reported a strong association between efflux pump gene expression and biofilm formation, a critical factor in recurrent infections. Our findings also highlight the significant presence of biofilm-associated genes, such as *mrkA*, further implicating their role in persistent infections in malignancy patients.

Virulence factors, particularly siderophores and fimbriae, were prevalent across the isolates. Iron acquisition, a critical factor for bacterial growth, is mediated by siderophores such as aerobactin, enterobactin, salmochelin, and yersiniabactin (Mirzaie and Ranjbar, 2021). Aerobactin, encoded by *iucABCD*, was detected in 49% of isolates, while enterobactin (*entABCDE*) was universally present, with individual gene carriage rates exceeding 90%. Salmochelin (*iroBCDN*) and yersiniabactin (*ybt*) were identified in 40 and 32% of isolates, respectively. Interestingly, cervical cancer patient samples isolates had the lowest prevalence of aerobactin (12.5%) and salmochelin (12.5%) genes. These siderophore gene carriage rates align with previous studies, underscoring their role as essential virulence factors for *K. pneumoniae* survival and pathogenesis (Mendes et al., 2023).

Virulence scoring, based on the system developed by Lam et al. (Kumabe and Kenzaka, 2014), showed that nearly 50% of isolates from malignant tumor patients scored ≥3, indicating high virulence potential. However, only 13% of cervical cancer patient samples isolates achieved this threshold, likely due to the smaller sample size. These virulence scores were higher than those reported by David J. Roach et al. (Jin et al., 2022), possibly reflecting the association between specific virulence factors and the malignancy-related infections studied here. The findings highlight the critical role of virulence determinants in the pathogenicity of *K. pneumoniae* in tumor patients.

Fimbriae play a crucial role in adhesion and biofilm formation, facilitating catheter-associated infections. The fimbrial genes *fimA*, *fimH*, and *mrkD*, encoding type 1 and type 3 fimbriae, were prevalent among the isolates. These fimbriae enhance *K. pneumoniae*’s ability to adhere to abiotic surfaces and form biofilms, critical for colonization and persistence in hospital settings (Roach et al., 2024; Arato et al., 2021). Wen-Ying Guo et al. (Schroll et al., 2010) reported that mrkABCDF and fimACDH operons, which encode these fimbriae, are often co-located with antibiotic resistance genes on plasmids, suggesting a linkage between virulence and resistance mechanisms. This co-occurrence further complicates treatment strategies for infections caused by these isolates.

Sequence typing involves analyzing the sequences of multiple housekeeping genes within bacterial genomes to determine genetic relationships between strains. In this study, whole-genome sequencing and database comparison identified 45 sequence types, with ST23 (15.3%) and ST45 (6.7%) being predominant. ST23, one of the major clones of hypervirulent *Klebsiella pneumoniae* (HV-KP) and a representative of the CC23 clonal lineage, is associated with high virulence and strong adaptability (Guo et al., 2022). Among ST23 isolates in this study, 87.5% were identified as HV-KP, consistent with previous reports. This suggests that most *K. pneumoniae* infections in cancer patients are caused by strains with high virulence and adaptability, contributing to recurrent, hard-to-cure infections and high mortality. Similarly, Siqin Zhang et al. (Lam et al., 2018) reported ST23 as the dominant type (38.7%) in *K. pneumoniae* strains causing pyogenic liver abscesses in southeastern China, aligning with our findings. In contrast, ST11 is commonly linked to hospital-acquired CRKP. Chen et al. (2024) reported ST11 as the predominant CRKP type (68.6%) in hematologic malignancy patients, while Yan Li et al. (Zhang et al., 2019) found ST11 in 66.7% of CRKP isolates from hospitalized patients across China, noting significant differences in antibiotic resistance between ST11 and non-ST11 strains. This study identified 8 CRKP isolates, with ST11 accounting for 25%, a lower proportion likely due to the limited sample size.

Phylogenetic analysis of 105 *K. pneumoniae* isolates revealed three distinct clades, with clustering patterns correlating to cancer types. *K. pneumoniae* strains isolated from lung, nasopharyngeal, liver, and cervical cancer patients were primarily grouped in the third clade, with liver and cervical cancer strains comprising 80 and 87.5%, respectively. This may reflect an association between infection dynamics and cancer type, as KP strains in respiratory tumors appear more transmissible. Previous studies have investigated KP strain evolution and dissemination. Liu et al. (2023); Li et al. (2024) analyzed the evolutionary relationships of 184 ST23-KP strains, identifying three clonal transmission events, mainly in ICUs, causing respiratory infections. These findings underscore the importance of preventing the hospital transmission of drug-resistant, hypervirulent KP strains. This study has several limitations. The relatively small sample size, particularly the limited number of cases for specific malignancy types, may constrain the generalizability of the findings. Furthermore, the absence of a non-cancer control group prevents definitive conclusions regarding whether the observed distribution of antimicrobial resistance genes and virulence factors is specific to cancer patients. Nevertheless, this work represents the first comprehensive genomic characterization of *K. pneumoniae* isolates from cancer patients in this region, providing valuable baseline data for future investigations. Subsequent studies should employ multi-center collaborations to expand the cohort and incorporate appropriately matched controls to verify and build upon these observations.

## 5 Conclusion

In conclusion, this study provides a comprehensive characterization of *K. pneumoniae* isolates from malignant tumor patients, revealing high levels of antibiotic resistance and virulence. The predominance of resistance genes, efflux pumps, siderophores, and fimbriae underscores the multifaceted mechanisms driving pathogenicity and persistence in these patients. The findings highlight the urgent need for targeted surveillance and effective infection control measures to manage *K. pneumoniae* infections in malignancy settings. Future studies should focus on larger sample sizes and explore the interplay between resistance and virulence mechanisms to develop novel therapeutic strategies.

## Data Availability

The raw sequencing data of 105 *Klebsiella pneumoniae* isolates generated in this study are publicly available in the NCBI BioProject database at: https://www.ncbi.nlm.nih.gov/bioproject/PRJNA1283480.

## Footnotes

^1^ https://pubmlst.org/efaecium/
